# Fixed and Adaptive Parallel Subgroup-Specific Design for Survival Outcomes: Power and Sample Size

**DOI:** 10.3390/jpm7040019

**Published:** 2017-12-04

**Authors:** Miranta Antoniou, Andrea L. Jorgensen, Ruwanthi Kolamunnage-Dona

**Affiliations:** 1 MRC North West Hub for Trials Methodology Research, Liverpool L69 3GL, UK; A.L.Jorgensen@liverpool.ac.uk (A.L.J.); Ruwanthi.Kolamunnage-Dona@liverpool.ac.uk (R.K.-D.); 2 Department of Biostatistics, Institute of Translational Medicine, University of Liverpool, Liverpool L69 3GL, UK

**Keywords:** biomarker, biomarker-guided trial design, clinical research design, personalized medicine, fixed design, adaptive design, sample size, simulation study, multiplicity issues

## Abstract

Biomarker-guided clinical trial designs, which focus on testing the effectiveness of a biomarker-guided approach to treatment in improving patient health, have drawn considerable attention in the era of stratified medicine with many different designs being proposed in the literature. However, planning such trials to ensure they have sufficient power to test the relevant hypotheses can be challenging and the literature often lacks guidance in this regard. In this study, we focus on the parallel subgroup-specific design, which allows the evaluation of separate treatment effects in the biomarker-positive subgroup and biomarker-negative subgroup simultaneously. We also explore an adaptive version of the design, where an interim analysis is undertaken based on a fixed percentage of target events, with the option to stop each biomarker-defined subgroup early for futility or efficacy. We calculate the number of events and patients required to ensure sufficient power in each of the biomarker-defined subgroups under different scenarios when the primary outcome is time-to-event. For the adaptive version, stopping probabilities are also explored. Since multiple hypotheses are being tested simultaneously, and multiple interim analyses are undertaken, we also focus on controlling the overall type I error rate by way of multiplicity adjustment.

## 1. Introduction

Biomarker-guided treatment is a rapidly developing area of medicine, where treatment choice is personalised according to an individual’s characteristics e.g., their genetic or other biomarker profile, or demographics. Stratified medicine, also known as ‘individualized medicine’, ‘personalized medicine’, or ‘precision medicine’ [1] refers to a population approach aiming to identify a particular subset of patients that benefit most (or least) from the treatment. 

A number of biomarker-guided trial designs have been proposed in the past decade, which test the effectiveness of a biomarker-guided approach to treatment with the aim of improving patient health. These designs can be classified into two categories; the adaptive designs thoroughly reviewed in Antoniou et al. [2] which allow adaptations in trial procedures and/or statistical procedures during the course of the study without undermining the validity and integrity of the trial; and the non-adaptive designs which are typically simpler but less flexible and discussed in depth in Antoniou et al. [3]. While such designs have been given significant attention in the literature, there are many challenges associated with their design, analysis and practical application, which need to be explored further and better understood. Key challenges include powering the study adequately, controlling the false-positive rate, and applying appropriate stopping probabilities.

In the current article we focus on the parallel subgroup-specific design [4,5,6] which can be explored more transparently to discuss the above challenges. The parallel subgroup-specific design is used to test the clinical hypothesis of treatment effect, evaluating the effect of the experimental treatment relative to a control treatment in both a biomarker-negative and a biomarker-positive subgroup separately. We also consider an adaptive version of the design, splitting the trial into two stages with the aim of stopping the study early for either positive or negative outcome. In this adaptive version, the first stage involves an interim analysis after the pre-specified percentage of events are achieved and a decision is made whether to stop the trial early for efficacy or futility, or to continue to the second stage of the study. Futility and efficacy are assessed by comparing the *p*-values of the observed test statistics produced at each stage of the design with pre-specified stopping boundaries. The role of an interim analysis in a clinical trial design is important as it might allow the experimental treatment to be made available earlier in case of positive results. We have conducted several simulation studies to evaluate a variety of scenarios. 

## 2. Methods and Findings

### 2.1. Parallel Subgroup-Specific Design

**Figure 1:**
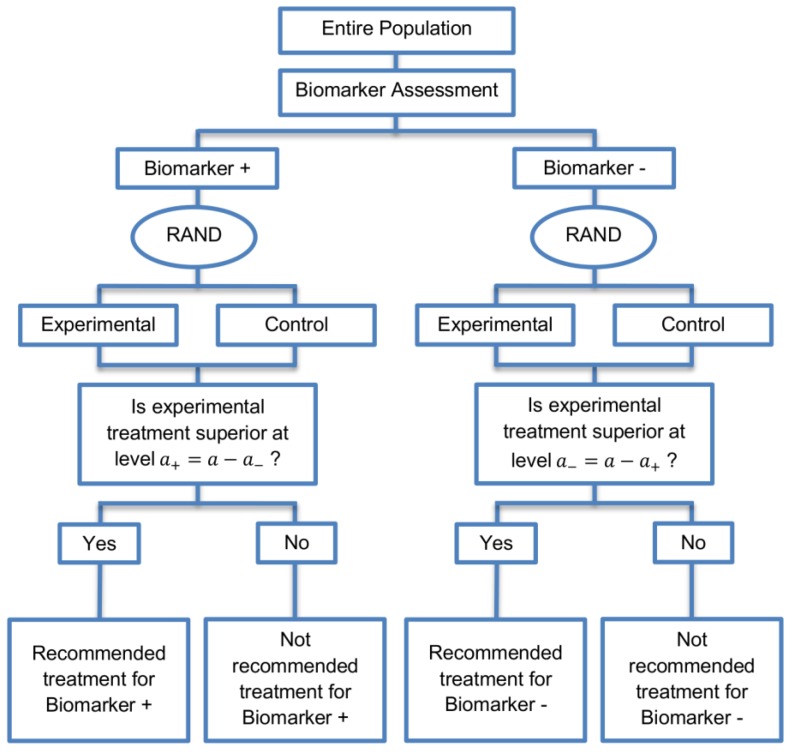
Parallel Subgroup-Specific design. “RAND” refers to randomization of patients. a refers to the overall significance level between the two biomarker subgroup tests such that $a=a_{-}+a_{+}$.

The parallel subgroup-specific design, a modified version of the marker stratified design, allows for the evaluation of treatment effects separately in the biomarker-positive subgroup and biomarker-negative subgroup at the same time [3]. While the marker-stratified design uses the overall significance level a for each biomarker-defined subgroup separately, the parallel subgroup-specific design controls the overall type I error rate by splitting the overall significance level a between the two biomarker subgroup tests such that $a=a_{-}+a_{+}$ [3]. A graphical illustration of this strategy is given in Figure 1.

All patients are screened for biomarker status (biomarker positive or biomarker negative) and then randomized to the experimental or control treatments in the two biomarker subgroups. Therefore, biomarker status acts as a stratification factor. Consequently, the trial is made up of four arms, i.e., biomarker-positive patients receiving either the experimental or the control treatment and biomarker-negative patients receiving either the experimental or the control treatment. A test for treatment effectiveness (Experimental treatment vs. Control treatment) can therefore be performed in each biomarker-defined subgroup separately.

Where a trial’s primary outcome is time to some specified event (e.g., time to death), the hypotheses being tested in the two biomarker subgroups if one assumes exponentially distributed times can be defined as follows:

(i) Hypothesis being tested (case of two-sided test) in the biomarker negative subgroup $H_{0,biom^{-}}:\;\log\left(\theta_{-}\right)=0$, where
  $$\theta_{-}=HR_{biom^{-}}=\frac{\lambda_{E-}}{\lambda_{C-}}$$
  denotes the hazard ratio, and $\lambda_{E-}$ and $\lambda_{C-}$ are the rate parameters of an exponential distribution for biomarker-negative patients receiving experimental treatment and control treatment respectively, and
(ii) Hypothesis being tested (case of two-sided test) in the biomarker positive subgroup $H_{0,biom^{+}}:\;\log\left(\theta_{+}\right)=0$, where
  $$\theta_{+}=HR_{biom^{+}}=\frac{\lambda_{E+}}{\lambda_{C+}}$$
  refers to the hazard ratio, and $\lambda_{E+}$ and $\lambda_{C+}$ are the rate parameters of an exponential distribution for biomarker-positive patients receiving experimental treatment and control treatment respectively.

#### 2.1.1. Sample Size Calculation for Time-to-Event Outcomes

For the purpose of undertaking power calculations for this design, we assume that the treatment effect will be tested using the log-rank test. The total number of events required for the parallel subgroup-specific design can be calculated by adding up the number of events required in each biomarker-defined subgroup. Following Mandrekar and Sargent [7], we assume 1:1 randomization, and therefore the required number of events for each biomarker-defined subgroup can be calculated by

(1) $$D_{j}=4\frac{{\left(z_{a_{j}}+z_{\beta }\right)}^{2}}{{\left[\log\left(\theta_{j}\right)\right]}^{2}}$$

where j denotes either the biomarker positive subgroup (j=+) or the biomarker negative subgroup (j=−), $z_{a_{j}}$, $z_{\beta }$ denote the upper $a_{j}$- and upper β-points respectively of a standard normal distribution and the required total number of events can be calculated by

(2) $$D=D_{-}+D_{+}=4\frac{{\left(z_{a_{-}}+z_{\beta }\right)}^{2}}{{\left[\log\left(\theta_{-}\right)\right]}^{2}}+4\frac{{\left(z_{a_{+}}+z_{\beta }\right)}^{2}\;}{{\left[\log\left(\theta_{+}\right)\right]}^{2}}$$

where $a_{-}$ and $a_{+}$, denote the type I error rates for biomarker-negative and biomarker-positive subgroup respectively such that $a_{-}+a_{+}=a$, and a is the nominal significance level (if one-sided e.g., a=0.025 in our case) and β corresponds to the type II error rate (it is common across the two subgroups). One-sided significance levels are used in situations where an alternative hypothesis specifies that the treatment benefit in the experimental group is greater than that of the control group. In case that a two-sided a is used (e.g., a=0.05), then (2) can be written as

$$D=D_{-}+D_{+}=4\frac{{\left(z_{a_{-}/2}+z_{\beta }\right)}^{2}}{{\left[\log\left(\theta_{-}\right)\right]}^{2}}+4\frac{{\left(z_{a_{+}/2}+z_{\beta }\right)}^{2}}{{\left[\log\left(\theta_{+}\right)\right]}^{2}}.$$

When more than one hypothesis for the assessment of experimental treatment efficacy is being tested, it is important to control the familywise error rate (FWER) by adjusting for multiplicity of testing to ensure that the probability to commit at least one type I error does not exceed the nominal significance level. To achieve this, a conservative Bonferroni correction method is often used where a is allocated between the test for the biomarker-negative subgroup and the test for the biomarker-positive subgroup either equally (i.e., a /2) or unequally, meaning that the significance levels assigned to each biomarker defined-subgroup then add up to the total significance levela .

The calculation of the total sample size needed for this study is based on the total number of events and the probability that a subject will get an event prior to the end of the study [8]. Therefore, the sample size required for subgroup *j* is,

(3) $$N_{j}=\frac{D_{j}}{{Pr}_{j}\left(event\right)}$$

where *j* refers to the biomarker-defined subgroup, ${Pr}_{j}\left(event\right)$ corresponds to the probability of observing an event in biomarker subgroup *j* which can be calculated by

$${Pr}_{j}\left(event\right)=\pi_{E}Pr_{Ej}\left(event\right)+\pi_{C}Pr_{Cj}\left(event\right),$$

with

$$\pi_{E}=\frac{R}{R+1}\;\mathrm{and}\;\pi_{C}=\frac{1}{R+1}.$$

$\pi_{E}$ and $\pi_{C}$ are the proportions of patients who are randomized to the experimental and control treatment arm respectively. *R* is the allocation ratio which is given by the sample size in experimental arm divided by the sample size in control arm. Here we assume equal allocation between treatment arms for each biomarker-defined subgroup [9]; hence R=1 and $\pi_{E}=\pi_{C}=0.5$. $Pr_{Ej}\left(event\right)$ and $Pr_{Cj}\left(event\right)$ are the probability of event in the experimental and control treatment arm respectively in subgroup *j*. If *i* now denotes treatment group (either experimental (*E*) or control (*C*)) and if one assumes exponentially distributed times, the probability of an event in treatment arm *i* of subgroup *j* can be calculated by

(4) $$Pr_{ij}\left(event\right)=1-\frac{1}{\left(\frac{\log\left(2\right)}{m_{ij}}\right)\times T_{j}}\times \left[e^{-\left(\frac{\log\left(2\right)}{m_{ij}}\right)\times \tau_{j}}-e^{-\left(\frac{\log\left(2\right)}{m_{ij}}\right)\times \left(T_{j}+\tau_{j}\right)}\right],$$

where $T_{j}$ corresponds to the length in months of the accrual period, during which an homogeneous Poisson entry process is assumed, of the biomarker-defined subgroup *j* and $\tau_{j}$ corresponds to the follow-up period of the biomarker-defined subgroup *j*. $m_{ij}$ denotes the median survival time of treatment arm *i* in biomarker-defined subgroup *j* where

(5) $$m_{E-}=\frac{m_{C-}}{\theta_{-}}$$

and

(6) $$m_{E+}=\frac{m_{C+}}{\theta_{+}}.$$

Equation (4) could be generalized to arbitrary, continuous survival functions.

Using the sample size of each biomarker-defined subgroup, the corresponding accrual rate (number of patients recruited per month) for subgroup *j* is $ar_{j}$ which can be calculated by

(7) $$ar_{j}=\frac{N_{j}}{T_{j}}.$$

#### 2.1.2. Simulation Study 1

The scope of this simulation study is to confirm that we can achieve the desirable power in each biomarker-defined subgroups under different simulation settings for a time-to-event outcome. We calculate the required number of events and patients for each biomarker-defined subgroup $\left(D_{j},N_{j}\right)$ from Equations (1) and (3). Different scenarios are considered by varying hazard ratios ($\theta_{-},\theta_{+}$) and significance levels $\left(a_{-},a_{+}\right)$. In our simulation study, we assume that the biomarker-negative patients have a worse treatment outcome as compared to the biomarker-positive subgroup. We assume outcome to be an adverse effect and so the assumed hazard ratio values <1 reflect the fact that the experimental treatment is superior to the control treatment in both biomarker subgroups. Further, the lower hazard ratio value assumed for a specific biomarker-defined subgroup reflects a greater treatment effect in that subgroup. Hence, in all scenarios of hazard ratios, we consider higher $\theta_{-}$ than $\theta_{+}$. More specifically, four scenarios of hazard ratios, i.e., (i) $\theta_{-}=0.6$ and $\theta_{+}=0.4$, (ii) $\theta_{-}=0.7$ and $\theta_{+}=0.5$, (iii) $\theta_{-}=0.8$ and $\theta_{+}=0.6$, and (iv) $\theta_{-}=0.9$ and $\theta_{+}=0.7$ and three scenarios of significance levels (i) $a_{-}=a_{+}=0.0125$, (ii) $a_{-}=0.015$ and $a_{+}=0.010$, and (iii) $a_{-}=0.010$ and $a_{+}=0.015$ are considered. We set the median survival time of biomarker-negative subgroup in control group ($m_{C-}$) in Equation (5) at five months and we calculate the corresponding median survival time for the experimental group in that subgroup. We set the median survival time of biomarker-positive subgroup in control group $\left(m_{C+}\right)$ in Equation (6) at ten months and we calculate the corresponding median survival time for the experimental group in that subgroup. Additionally, we set the type II error rate at 20%, i.e., β=0.2 in Equation (1) which corresponds to 80% power (i.e., 1−β=0.8), length of accrual period $\left(T_{j}\right)$ in Equation (4) at 18 months and length of follow-up time $\left(\tau_{j}\right)$ in Equation (4) at 12 months for each biomarker-defined subgroup. Study entry times and event times for each biomarker-defined subgroup are generated as described below.

The time of study entry for participants in each biomarker-defined subgroup is modeled with a uniform distribution for entry times. More precisely, the entry times of patients recruited into the biomarker negative subgroup in the first month are assumed by randomly generating $ar_{-}$ (the accrual rate) numbers from U~Unif[0,1]. Similarly, $ar_{+}$ numbers are generated from U~Unif[0,1] to obtain study entry times of patients recruited into the biomarker positive subgroup during the first month. To obtain study entry times for those in the biomarker negative and biomarker positive subgroups during the second month, a further $ar_{-}$ and $ar_{+}$ numbers respectively are randomly generated from U~Unif[1,2]. The accrual continues until the assumed accrual period $T_{j}$. Thus, in the $T_{j}{}^{th}$ month, study entry times are generated from $U\sim Unif\left[T_{j}-1,T_{j}\right]$. At the end of the accrual period $N_{+}$ and $N_{-}$ participants in total have been recruited.

Event times are generated from an exponential distribution assuming hazard rate $\lambda_{ij}$ for jth biomarker-defined subgroup receiving treatment *i*. The values of $\lambda_{ij}$ can be determined by

$$S_{ij}\left(m_{ij}\right)=\exp\left(-\lambda_{ij}\times m_{ij}\right)=0.5,$$

where $m_{ij}$ are corresponding median survival times, and $S_{ij}\left(m_{ij}\right)$ is the exponential median survival probability for subgroup *j* and treatment *i*. By solving $S_{ij}\left(m_{ij}\right)$ for $\lambda_{ij}$ gives

$$\lambda_{ij}=\frac{\ln2}{m_{ij}}.$$

We assume patients are not lost to follow-up during the study, and hence any censoring in both biomarker-defined subgroups is due to the event occurring after a cutofftime. The cutoff time refers to the time after study start at which a pre-specified number of events $D_{j}$ for each biomarker-defined subgroup has been reached. A time $t_{ij}$ (i.e., sum of accrual time and follow-up time) is generated for each patient, and if $t_{ij}$ is greater than the cutoff time then it is assumed that the patient encountered the event at $t_{ij}$, otherwise the patient’s event time is censored at $t_{ij}$.

One-sided *p*-values for treatment effect in each biomarker-defined subgroup are computed using the log-rank test. One-sided *p*-values are considered because we assume that the treatment benefit in the experimental group is greater than that in the control group.

#### 2.1.3. Results from Simulation Study 1

**Figure 2:**
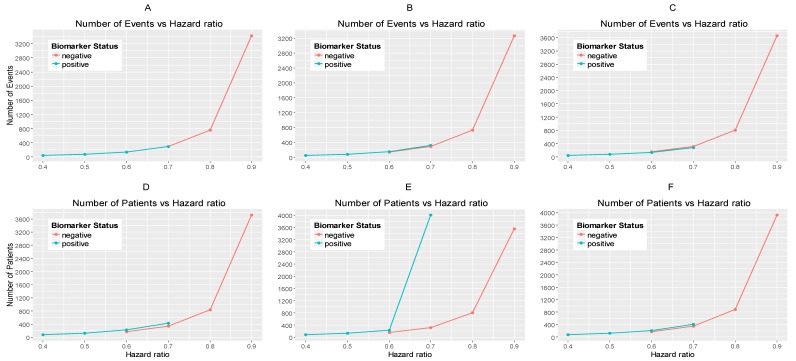
(**A**–**C**) represent the required number of events and (**D**–**F**) represent the required number of patients of each biomarker-defined subgroup which achieve 80% power versus the corresponding hazard ratio for each of the three scenarios of significance levels. (**A**,**D**) correspond to the significance levels $a_{-}=a_{+}=0.0125$, (**B**,**E**) correspond to the significance levels $a_{-}=0.015$ and $a_{+}=0.010$ and (**C**,**F**) correspond to the significance levels $a_{-}=0.010$ and $a_{+}=0.015$.

The results are drawn from 10,000 iterations. The simulated power of each biomarker-defined subgroup is preserved approximately at 80% across all scenarios of hazard ratios and significance levels. The accrual rates and the number of events and patients to reach the nominal level of power (80%) corresponding to different scenarios of hazard ratios and significance levels are presented in Table S1 provided in Supplementary Materials, File S. The power for each biomarker-defined subgroup yielded from the simulation study is also presented. Figure 2A–C illustrates the required number of events for each biomarker-defined subgroup versus the corresponding hazard ratio for each of the three scenarios of significance levels. Figure 2D–F illustrates the required number of patients of each biomarker-defined subgroup versus the corresponding hazard ratio for each of the three scenarios of significance levels. As expected, the number of events and therefore the sample size required for each biomarker-defined subgroup increases with the increase of the corresponding hazard ratio at the same significance level. Furthermore, at each scenario of hazard ratio, we can achieve a smaller sample size and necessary number of events for each biomarker-defined subgroup with a larger significance level (for example, when HR scenario (i) $\theta_{-}=0.6$ and $\theta_{+}=0.4$, and when $a_{+}=$ 0.015, we achieve the smallest necessary number of events and sample sizes).

From Table S1, and more clearly from Figure 2A–F, it can be seen that for each scenario of hazard ratios, the required number of events and patients in the biomarker-negative subgroup is greater than in biomarker-positive subgroup.

**Figure 3:**
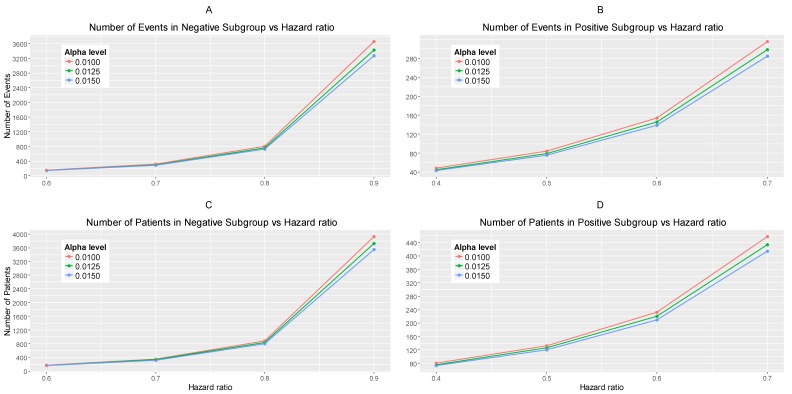
(**A**,**B**) represent the required number of events and (**C**,**D**) represent the required number of patients which achieve 80% power versus the hazard ratio in each of the three scenarios of significance levels for each biomarker-defined subgroup separately. (**A**,**C**) correspond to the biomarker-negative subgroup and the following significance levels: (i) $a_{-}=0.0125$, (ii) $a_{-}=0.015$, and (iii) $a_{-}=0.010$. (**B**,**D**) correspond to the biomarker-positive subgroup and the following significance levels: (i) $a_{+}=0.0125$, (ii) $a_{+}=0.010$, and (iii) $a_{+}=0.015$.

Figure 3A,B represents the required number of events which achieve 80% power versus the hazard ratio for each of the three scenarios of significance levels in each biomarker-defined subgroup separately. Figure 3C,D represents the required number of patients which achieve 80% power versus the hazard ratio for each of the three scenarios of significance levels in each biomarker-defined subgroup separately. The corresponding numerical results are presented in Table S1.

It can be seen that for all scenarios of hazard ratios, the highest value of the number of events and patients in the biomarker-negative subgroup and in the biomarker-positive subgroup is given by $a_{-}=0.010$ and $a_{+}=0.010$ for negative and positive patients respectively and the lowest value is given by $a_{-}=0.015$ and $a_{+}=0.015$ for negative and positive patients respectively.

### 2.2. An Adaptive Version of the Parallel Subgroup-Specific Design

**Figure 4:**
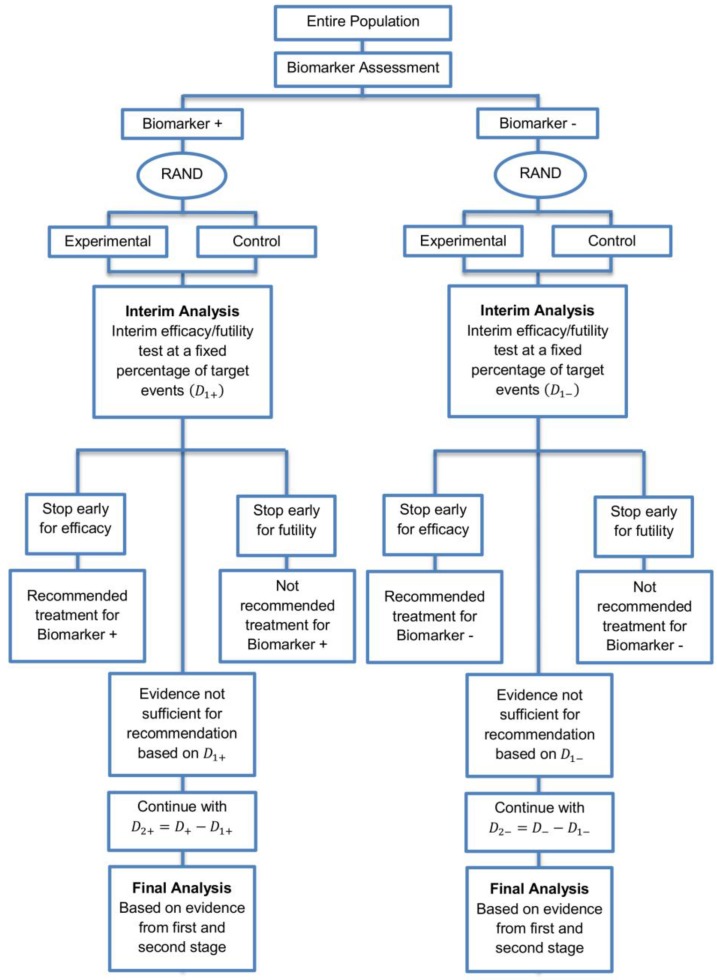
Adaptive parallel subgroup-specific design. “RAND” refers to randomization of patients. $D_{1+}$ and $D_{1-}$ correspond to the target number of events of the biomarker-positive subgroup and biomarker-negative subgroup respectively at the first stage of the study. $D_{+}$ and $D_{-}$ correspond to the total required number of events of the biomarker-positive subgroup and biomarker-negative subgroup respectively which are planned according to the non-adaptive approach. $D_{2+}$ and $D_{2-}$ correspond to the number of events of the biomarker-positive subgroup and biomarker-negative subgroup respectively at the second stage of the study.

We explore a two-stage adaptive design starting with the parallel assessment of treatment effect in each biomarker-defined subgroup. In the first stage an interim analysis is included where each biomarker-defined sub-group can stop early for futility or efficacy. The interim analysis is based on a fixed and pre-specified percentage of target events. In case that we do not stop after the first stage due to early efficacy or futility, the trial continues to the second stage, testing the efficacy of the experimental treatment in each biomarker-defined subgroup separately. A graphical illustration of this strategy is given in Figure 4.

Adaptive designs differ from fixed designs in that they permit the performance of interim analyses during the course of the study leading to adaptations of hypotheses which are under investigation. Results from interim analyses are used to make a decision regarding the trial. Several sources of multiplicity problems can arise in the conduct of adaptive trial designs resulting in the inflation of the overall type I error rate (probability of a false positive result). One of the sources of type I error inflation is the adaptation of design and analysis features with combination of information across trial stages [10]. Hence, alpha-adjustment (i.e., adjustment of the alpha level at each interim analysis so that the overall type I error rate remains at the desired level) is needed so that the overall type I error rate remains under control. A variety of methods for the control of type I error rate in adaptive designs have been proposed which are thoroughly summarized by Chang [11]. Our study is based on a flexible and general approach to adaptive designs for a-adjustment proposed by Chang [12] in which the test statistic of the final analysis is defined as the sum of the unadjusted stagewise *p*-values ($p_{l}$). More precisely, at the *k*th stage of an adaptive design, the test statistic which can be viewed as cumulative evidence against the null is given by

$${T^{\prime }}_{k}=\sum_{l=1}^{k}p_{l},\;k=1,\ldots ,L=:“maximum\;stage”.$$

Before conducting the two-stage design, pre-specification of stopping rules and stopping boundaries for efficacy and futility are needed. Stopping probabilities (i.e., rejection probabilities) which are calculated based on the stopping boundaries, are essential operating characteristics of adaptive designs and they are classified into two types. The first type is the so-called ‘efficacy stopping probability’, which refers to the unconditional probability of rejecting the null hypothesis of no treatment effect, thus the trial stops in order to claim efficacy. The second type is the so-called ‘futility stopping probability’, which refers to the unconditional probability of not rejecting the null hypothesis of no treatment effect, thus the trial stops in order to claim futility. Hence, the following stopping boundaries should be chosen: (i) the early efficacy stopping boundaries in stage 1, i.e., $\epsilon_{1-}$ and $\epsilon_{1+}$ for biomarker-negative and biomarker-positive patients respectively, (ii) the early futility stopping boundaries in stage 1, i.e., $b_{1-}$ and $b_{1+}$ for biomarker-negative and biomarker-positive patients respectively, and (iii) the final efficacy stopping boundaries, $\epsilon_{2-}$ and $\epsilon_{2+}$ for biomarker-negative and biomarker-positive patients respectively.

If there is prior belief that the experimental treatment is of strong benefit to patients, then the trial should be designed without early futility stopping (i.e., we need to set a larger value for $b_{1-}$ and/or $b_{1+}$). When early efficacy stopping is allowed (e.g., to allow possibility of making treatment available to patients earlier or to allow possibility of unnecessary treatment exposure or unnecessary trial costs), then the trial should be designed with a large value of $\epsilon_{1-}$ and/or $\epsilon_{1+}$.

After the appropriate choice of $\epsilon_{1-}$, $\epsilon_{1+}$ and $b_{1-}$, $b_{1+}$, we can solve for the final efficacy stopping boundaries, i.e., $\epsilon_{2-}$, $\epsilon_{2+}$ with reference to Chang et al.’s method, based on the sum of *p*-values. More precisely, in a clinical trial with *k* interim analyses, the stopping boundary can be derived by calculating the rejection probability under the null hypothesis which takes into account the stopping rules described below. The rejection probability at the *k*th stage is defined by $\psi_{k}\left(\epsilon_{k}\right)$, where

(8) $$\psi_{k}\left(t^{\prime }\right)=\mathrm{Pr}(\epsilon_{1}<{T^{\prime }}_{1}<b_{1},\ldots ,\epsilon_{k-1}<{T^{\prime }}_{k-1}<b_{k-1},\;{T^{\prime }}_{k}<t^{\prime })=\int_{\epsilon_{1}}^{b_{1}}\ldots \int_{\epsilon_{k-1}}^{b_{k-1}}\int_{-\infty }^{t^{\prime }}f_{{T^{\prime }}_{1}\ldots {T^{\prime }}_{k}}\left({t^{\prime }}_{1},\ldots {t^{\prime }}_{k}\right)d{t^{\prime }}_{k}d{t^{\prime }}_{k-1}\ldots d{t^{\prime }}_{1}$$

where $t^{\prime }\geq 0$, ${t^{\prime }}_{l}\left(l=1,\ldots ,k\right)$ is the test statistic at the lth stage, and $f_{{T^{\prime }}_{1}\ldots {T^{\prime }}_{k}}$ is the joint probability density function of ${T^{\prime }}_{1},\ldots {T^{\prime }}_{k}$. The stopping rules for futility can be either binding or non-binding. In the non-binding rule the possibility of stopping early for futility will not be considered in the decision of the efficacy stopping boundary whereas in the binding category the futility rule is taken into account when making inference. As it is stated by Chang [11], the regulatory bodies currently adopt the non-binding futility rule in order to ensure that the familywise type I error rate is controlled regardless of whether a decision is made to continue the trial despite a futility boundary being crossed. For this reason, we set $b_{1-}=\epsilon_{2-}$ and $b_{1+}=\epsilon_{2+}$. Based on Equation (8), according to Chang [12] the final efficacy stopping boundaries can be found by

$$\epsilon_{2}=\sqrt{\left(a-\epsilon_{1}\right)}+\epsilon_{1},$$

where $\epsilon_{1}<a$ and a refers to the level of significance. In our case, the final efficacy stopping boundaries for each biomarker-defined subgroup with non-binding futility rule can be found by the following formulations,

$$\epsilon_{2-}=\sqrt{\left(a_{-}-\epsilon_{1-}\right)}+\epsilon_{1-},$$

$$\epsilon_{2+}=\sqrt{\left(a_{+}-\epsilon_{1+}\right)}+\epsilon_{1+},$$

where $\epsilon_{1-}<a_{-}$ and $\epsilon_{1+}<a_{+}$.

For the biomarker-negative subgroup of the two-stage adaptive design which tests the efficacy of the experimental treatment, the stopping rules are the following,

$$\mathrm{Stage}\;1:\{\begin{array}{c} \mathrm{Reject}\;\mathrm{the}\;\mathrm{null}\;\mathrm{hypothesis}\;\left(\mathrm{stop}\;\mathrm{for}\;\mathrm{efficacy}\right)\;\mathrm{if}\;{T^{\prime }}_{1-}\leq \epsilon_{1-}\\ \mathrm{Do}\;\mathrm{not}\;\mathrm{reject}\;\mathrm{the}\;\mathrm{null}\;\mathrm{hypothesis}\;\left(\mathrm{stop}\;\mathrm{for}\;\mathrm{futility}\right)\;\mathrm{if}\;{T^{\prime }}_{1-}>b_{1-}\\ \mathrm{Continue}\;\mathrm{to}\;\mathrm{the}\;\mathrm{second}\;\mathrm{stage}\;\mathrm{if}\;\epsilon_{1-}<{T^{\prime }}_{1-}\leq b_{1-} \end{array}$$

where $0<\epsilon_{1-}<b_{1-}\leq 1$ and ${T^{\prime }}_{1-}$ refers to the log-rank test statistic in the biomarker-negative subgroup at the first stage of the study,

$$\mathrm{Stage}\;2:\{\begin{array}{c} \mathrm{Reject}\;\mathrm{the}\;\mathrm{null}\;\mathrm{hypothesis}\;\left(\mathrm{stop}\;\mathrm{for}\;\mathrm{efficacy}\right)\;\mathrm{if}\;{T^{\prime }}_{2-}\leq \epsilon_{2-}\\ \mathrm{Do}\;\mathrm{not}\;\mathrm{reject}\;\mathrm{the}\;\mathrm{null}\;\mathrm{hypothesis}\;\left(\mathrm{stop}\;\mathrm{for}\;\mathrm{futility}\right)\;\mathrm{if}\;{T^{\prime }}_{2-}>\epsilon_{2-} \end{array}$$

where ${T^{\prime }}_{2-}$ refers to the log-rank test statistic in the biomarker-negative subgroup at the second stage of the study.

For the biomarker-positive subgroup of the two-stage adaptive design which tests the efficacy of the experimental treatment, the stopping rules are the following,

$$\mathrm{Stage}\;1:\{\begin{array}{c} \mathrm{Reject}\;\mathrm{the}\;\mathrm{null}\;\mathrm{hypothesis}\;\left(\mathrm{stop}\;\mathrm{for}\;\mathrm{efficacy}\right)\;\mathrm{if}\;{T^{\prime }}_{1+}\leq \epsilon_{1+}\\ \mathrm{Do}\;\mathrm{not}\;\mathrm{reject}\;\mathrm{the}\;\mathrm{null}\;\mathrm{hypothesis}\;\left(\mathrm{stop}\;\mathrm{for}\;\mathrm{futility}\right)\;\mathrm{if}\;{T^{\prime }}_{1+}>b_{1+}\\ \mathrm{Continue}\;\mathrm{to}\;\mathrm{the}\;\mathrm{second}\;\mathrm{stage}\;\mathrm{if}\;\epsilon_{1+}<{T^{\prime }}_{1+}\leq b_{1+} \end{array}$$

where $0<a_{1+}<b_{1+}\leq 1$ and ${T^{\prime }}_{1+}$ refers to the log-rank test statistic in the biomarker-positive subgroup at the first stage of the study,

$$\mathrm{Stage}\;2:\{\begin{array}{c} \mathrm{Reject}\;\mathrm{the}\;\mathrm{null}\;\mathrm{hypothesis}\;\left(\mathrm{stop}\;\mathrm{for}\;\mathrm{efficacy}\right)\;\mathrm{if}\;{T^{\prime }}_{2+}\leq \epsilon_{2+}\\ \mathrm{Do}\;\mathrm{not}\;\mathrm{reject}\;\mathrm{the}\;\mathrm{null}\;\mathrm{hypothesis}\;\left(\mathrm{stop}\;\mathrm{for}\;\mathrm{futility}\right)\;\mathrm{if}\;{T^{\prime }}_{2+}>\epsilon_{2+} \end{array}$$

where ${T^{\prime }}_{2+}$ refers to the log-rank test statistic in the biomarker-positive subgroup at the second stage of the study.

We now assume the interim fraction or information fraction for the biomarker-negative subgroup to be $f_{-}$ which refers to a specific proportion of the required total number of events in the biomarker-negative subgroup, and the interim fraction for the biomarker-positive subgroup be $f_{+}$ which refers to a specific proportion of the required total number of events in the biomarker-positive subgroup. Using these interim fractions, we calculate the target number of events for each subgroup at the interim stage (stage 1), to be:

$$D_{1-}=D_{-}\times f_{-},$$

$$D_{1+}=D_{+}\times f_{+},$$

for negative and positive patients respectively. The log-rank test statistics of each biomarker-defined subgroup at the first stage (interim analysis) are based on $D_{1-}$, $D_{1+}$ and given by

$${T^{\prime }}_{1-}=\sqrt{\frac{{\hat{D}}_{1-}}{4}}\times \left[\log\left({\hat{\theta }}_{-}\right)\right]\sim N\left(\sqrt{\frac{D_{1-}}{4}}\times \left[\log\left(\theta_{-}\right)\right],\;1\right),$$

$${T^{\prime }}_{1+}=\sqrt{\frac{{\hat{D}}_{1+}}{4}}\times \left[\log\left({\hat{\theta }}_{+}\right)\right]\sim N\left(\sqrt{\frac{D_{1+}}{4}}\times \left[\log\left(\theta_{+}\right)\right],\;1\right),$$

for the biomarker-negative subgroup and biomarker-positive subgroups respectively. One-sided *p*-values corresponding to the observed values ${t^{\prime }}_{1-}$ and ${t^{\prime }}_{1+}$ of the test statistics of each biomarker-defined subgroup in stage 1 are given by

$$p_{1-}=Pr\left({T^{\prime }}_{1-}\geq {t^{\prime }}_{1-}|H_{0,biom^{-}}\right),$$

$$p_{1+}=Pr\left({T^{\prime }}_{1+}\geq {t^{\prime }}_{1+}|H_{0,biom^{+}}\right),$$

for the biomarker-negative subgroup and biomarker-positive subgroups respectively.

In the first interim analysis, the test statistic is equal to the *p*-value at stage 1; hence in our simulation study we proceed with the following rules: If $p_{1-}>b_{1-}$ or/and $p_{1+}>b_{1+}$ then the study which is testing the efficacy of the experimental treatment in biomarker-negative subgroup and/or biomarker-positive subgroup is stopped for futility at stage 1. If $p_{1-}\leq \epsilon_{1-}$ and/or $p_{1+}\leq \epsilon_{1+}$ then the study which is testing the efficacy of the experimental treatment in biomarker-negative subgroup and/or biomarker-positive subgroup is stopped for efficacy at stage 1. Otherwise, if $\epsilon_{1-}<p_{1-}\leq b_{1-}$ and/or if $\epsilon_{1+}<p_{1+}\leq b_{1+}$, the study which is testing the treatment effect in each biomarker-defined subgroup continues to the second stage.

The log-rank test statistics of each biomarker-defined subgroup at the second stage of the study are given by

$${T^{\prime \prime }}_{2-}=\sqrt{\frac{\left(\hat{D_{-}-D_{1-}}\right)}{4}}\times \left[\log\left({\hat{\theta }}_{-}\right)\right]\sim N\left(\sqrt{\frac{\left(D_{-}-D_{1-}\right)}{4}}\times \left[\log\left(\theta_{-}\right)\right],\;1\right),$$

$${T^{\prime \prime }}_{2+}=\sqrt{\frac{\left(\hat{D_{+}-D_{1+}}\right)}{4}}\times \left[\log\left({\hat{\theta }}_{+}\right)\right]\sim N\left(\sqrt{\frac{\left(D_{+}-D_{1+}\right)}{4}}\times \left[\log\left(\theta_{+}\right)\right],\;1\right),$$

for the biomarker-negative subgroup and biomarker-positive subgroups respectively. One-sided p-values corresponding to the observed values ${t^{\prime \prime }}_{2-}$ and ${t^{\prime \prime }}_{2+}$ of the test statistics of each biomarker-defined subgroup in stage 2 are given by

$$p_{2-}=Pr\left({T^{\prime \prime }}_{2-}\geq {t^{\prime \prime }}_{2-}|H_{0,biom^{-}}\right),$$

$$p_{2+}=Pr\left({T^{\prime \prime }}_{2+}\geq {t^{\prime \prime }}_{2+}|H_{0,biom^{+}}\right),$$

for the biomarker-negative subgroup and biomarker-positive subgroup respectively. The test statistic of the final analysis for each biomarker-defined subgroup is based on the sum of stagewise *p*-values and can be given by

$${T^{\prime }}_{-}=p_{1-}+p_{2-},$$

$${T^{\prime }}_{+}=p_{1+}+p_{2+},$$

for the biomarker-negative subgroup and biomarker-positive subgroup respectively.

#### 2.2.1. Simulation Study 2

To investigate the effect of introducing an adaptive element to our study design, we have conducted a second simulation study which is performed by using the R statistical software (R Foundation for Statistical Computing, Vienna, Austria). To do this we assume the same total number of events and patients as we did for Simulation Study 1 (Section 2.1.2) where our design was not adaptive, therefore making the same assumptions regarding significance levels for biomarker-negative and biomarker-positive subgroups $\left(a_{-},\;a_{+}\right)$, hazard ratios ($\theta_{-},\theta_{+}$), median survival time in control group ($m_{C-},\;m_{C+}$), accrual period ($T_{-},\;T_{+}$) and follow-up period ($\tau_{-},\;\tau_{+}$) as we did previously. Therefore we assume accrual time $\left(T_{j}\right)$ to be 18 months for both subgroups, follow-up time $\left(\tau_{j}\right)$ to be 12 months for both subgroups and consider the following four different scenarios for the hazard ratios, (i) $\theta_{-}=0.6$ and $\theta_{+}=0.4$, (ii) $\theta_{-}=0.7$ and $\theta_{+}=0.5$, (iii) $\theta_{-}=0.8$ and $\theta_{+}=0.6$, and (iv) $\theta_{-}=0.9$ and $\theta_{+}=0.7$. For each scenario of hazard ratios we again assume three different scenarios for significance levels for the biomarker-negative and biomarker-positive subgroups, i.e., (i) $a_{-}=a_{+}=0.0125$, (ii) $a_{-}=0.015$ and $a_{+}=0.010$, and (iii) $a_{-}=0.010$ and $a_{+}=0.015$. For each hazard ratios and significance level combination explored previously, we test the implication of different percentages of the information fraction. The different information fractions considered are as follows: (i) $f_{-}=f_{+}=25\%$, (ii) $f_{-}=f_{+}=50\%$, and (iii) $f_{-}=f_{+}=75\%$. Our aim is to explore the impact of these different information fractions on study power as well as on the stopping probabilities for futility $(FSP_{j}$) and efficacy $(ESP_{j}).$

In our simulation study for all the scenarios of hazard ratios for each biomarker-defined subgroup we used a high value of early efficacy stopping boundaries, i.e., $\epsilon_{1+},\;\epsilon_{1-}$ and thus a high value of early futility stopping boundaries, i.e., $b_{1+},\;b_{1-}$ as it is believed that the experimental treatment is promising. Thus, for the three cases of significance levels for each biomarker-defined subgroup we have set the following stopping boundaries:

(i) when $a_{-}=a_{+}=0.0125,$
  for $\epsilon_{1+}=0.0080$ we get $b_{1+}=\epsilon_{2+}=\sqrt{\left(a_{+}-\epsilon_{1+}\right)}+\epsilon_{1+}=0.1029$,
  for $\epsilon_{1-}=0.0070$ we get $b_{1-}=\epsilon_{2-}=\sqrt{\left(a_{-}-\epsilon_{1-}\right)}+\epsilon_{1-}=0.1129$,
(ii) when $a_{-}=0.015$ and $a_{+}=0.010$,
  for $\epsilon_{1+}=0.0080$ we get $b_{1+}=\epsilon_{2+}=\sqrt{\left(a_{+}-\epsilon_{1+}\right)}+\epsilon_{1+}=0.0527$,
  for $\epsilon_{1-}=0.0070$ we get $b_{1-}=\epsilon_{2-}=\sqrt{\left(a_{-}-\epsilon_{1-}\right)}+\epsilon_{1-}=0.0964$,
(iii) when $a_{-}=0.010$ and $a_{+}=0.015$,
  for $\epsilon_{1+}=0.0080$ we get $b_{1+}=\epsilon_{2+}=\sqrt{\left(a_{+}-\epsilon_{1+}\right)}+\epsilon_{1+}=0.0917$,
  for $\epsilon_{1-}=0.0070$ we get $b_{1-}=\epsilon_{2-}=\sqrt{\left(a_{-}-\epsilon_{1-}\right)}+\epsilon_{1-}=0.0618$.

In all cases we have used a slightly lower value for $\epsilon_{1-}$ (i.e., 0.007) assuming that it is believed that the experimental treatment is less promising in biomarker-negative subgroup as compared to the biomarker-positive subgroup.

Different values of stopping boundaries could be used for each assumed scenario of hazard ratios and significance levels based on how promising the experimental treatment seems to be in each subgroup. However, for simplicity, in our study we set only one value of $\epsilon_{1+}$ for biomarker-positive subgroup and only one value of $\epsilon_{1-}$ for biomarker-negative subgroup in all cases of hazard ratios.

The general efficiency related to the cost and time of the trial can be seen from the expected number of events and expected sample size of the trial which are calculated by using the futility and efficacy stopping probabilities. The expected sample size is defined by Chang [11] as a function of the effect size and its uncertainty, which are unknown. Hence, apart from the stopping probabilities and power, this simulation study also provides the average expected number of events for each biomarker-defined subgroup ($D_{-}^{exp},\;D_{+}^{exp}$). Based on the futility ($FSP_{j}$) and efficacy stopping probabilities ($ESP_{j}$), we also calculate the average expected sample size for each biomarker-defined subgroup, i.e.,

$$N_{-}^{exp}=\left[\left(ESP_{-}+FSP_{-}\right)\times N_{1-}\right]+\left\{\left[1-\left(ESP_{-}+FSP_{-}\right)\right]\times N_{-}\right\},$$

$$N_{+}^{exp}=\left[\left(ESP_{+}+FSP_{+}\right)\times N_{1+}\right]+\left\{\left[1-\left(ESP_{+}+FSP_{+}\right)\right]\times N_{+}\right\},$$

for negative and positive patients respectively. Assuming that we have constant accrual, we can calculate the expected duration of the trial for testing the treatment effect in each biomarker-defined subgroup, i.e.,

$$T_{-}^{exp}=\left[\left(ESP_{-}+FSP_{-}\right)\times \left(T_{-}+\tau_{-}\right)\times f_{-}\right]+\left\{\left[1-\left(ESP_{-}+FSP_{-}\right)\right]\times \left(T_{-}+\tau_{-}\right)\right\},$$

$$T_{+}^{exp}=\left[\left(ESP_{+}+FSP_{+}\right)\times \left(T_{+}+\tau_{+}\right)\times f_{+}\right]+\left\{\left[1-\left(ESP_{+}+FSP_{+}\right)\right]\times \left(T_{+}+\tau_{+}\right)\right\},$$

for negative and positive patients respectively.

#### 2.2.2. Results from Simulation Study 2

Table 1 in the main manuscript and Tables S2–S4 in Supplementary Materials (File S) provide the simulation results drawn from 10,000 iterations for each scenario of hazard ratios and significance levels, for each different assumed percentage of information fraction, i.e., (i) $f_{-}=f_{+}=25\%$, (ii) $f_{-}=f_{+}=50\%$, and (iii) $f_{-}=f_{+}=75\%$. We report the expected number of events and patients, expected total study duration, futility stopping probability, efficacy stopping probability and total power of the study in additional to the required number of patients and events (as presented in Table S1 for the fixed Parallel Subgroup-Specific design).

From Table 1 and Tables S2–S4, it can be seen that the futility stopping probability of each biomarker-defined subgroup at each significance level decreases when the percentage of information fraction increases. On the contrary, the efficacy stopping probability, the total power of the study, the sample size and the number of events increase with the increase of the information fraction.

**Figure 5:**
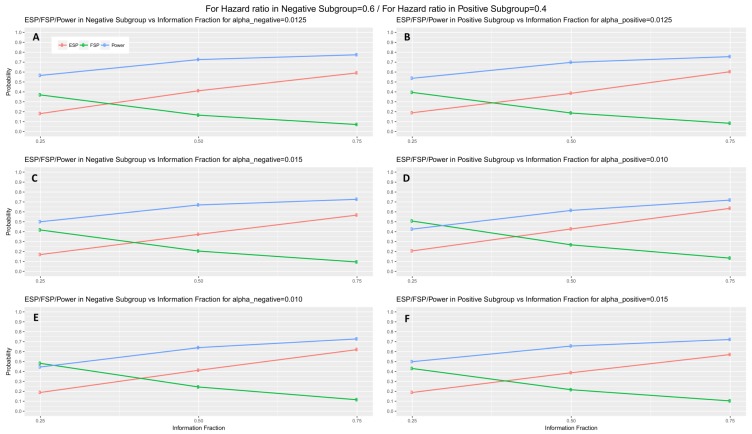
Efficacy stopping probability, futility stopping probability and power of a two-stage design versus the interim fraction (25%, 50%, 75%) in each biomarker-defined subgroup for scenario 1 of hazard ratios. Each row of graphs represents the different probabilities versus the interim fraction of each biomarker-defined subgroup when (i) $a_{-}=a_{+}=0.0125$ (**A**,**B**), (ii) $a_{-}=0.015$ and $a_{+}=0.010$ in (**C**,**D**), and (iii) $a_{-}=0.010$ and $a_{+}=0.015$ in (**E**,**F**) respectively.

When the interim fraction is set to 25% of the required total number of events, the simulation results indicate that the trial is underpowered. When the interim fraction is based on 50% of the required total number of events we still do not have a gain in power compared to the nominal level 80%, however, it achieves approximately 73% and 70% power in biomarker-negative and biomarker-positive subgroup respectively in all scenarios of hazard ratios when $a_{-}=a_{+}=0.0125$. When the interim fraction is based on 75% of the required total number of events, we can achieve higher level of power, i.e., approximately 77% and 76% power in biomarker-negative and biomarker-positive subgroup respectively in all scenarios of hazard ratios when $a_{-}=a_{+}=0.0125$. These results for the first scenario of hazard ratios are graphically represented in Figure 5 (main manuscript). The results for the remaining scenarios of hazard ratios are graphically represented in Figures S1–S3 in Supplementary Materials, File S.

**Figure 6:**
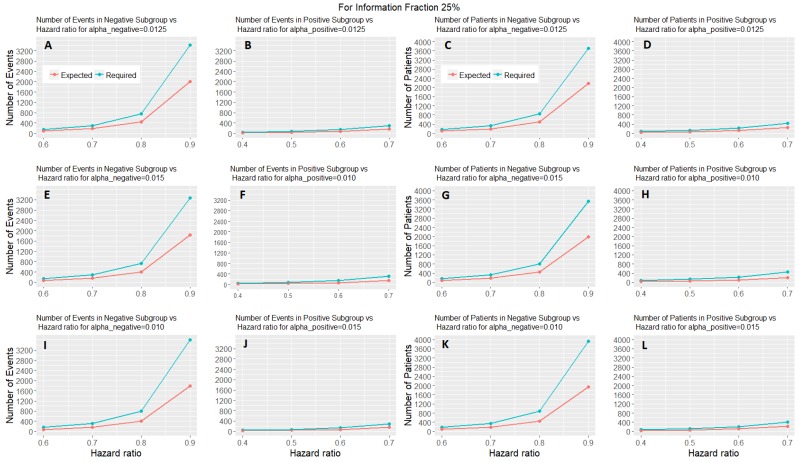
Expected number of events and patients in two-stage design and required number of events and patients in one-stage design for each biomarker-defined subgroup versus the hazard ratios of each biomarker-defined subgroup when the interim fraction is 25%. The first two graphical representations in each row of graphs represent the number of events versus the hazard ratio of each biomarker-defined subgroup when (i) $a_{-}=a_{+}=0.0125$ in (**A**,**B**), (ii) $a_{-}=0.015$ and $a_{+}=0.010$ in (**E**,**F**), and (iii) $a_{-}=0.010$ and $a_{+}=0.015$ in (**I**,**J**) respectively. The remaining graphical representations in each row of graphs represent the number of patients versus the hazard ratio of each biomarker-defined subgroup when (i) $a_{-}=a_{+}=0.0125$ in (**C**,**D**), (ii) $a_{-}=0.015$ and $a_{+}=0.010$ in (**G**,**H**), and (iii) $a_{-}=0.010$ and $a_{+}=0.015$ in (**K**,**L**), respectively.

Figure 6 shows the expected number of events and patients of the adaptive parallel subgroup-specific design and the required number of events and patients of the fixed parallel subgroup-specific design for each biomarker-defined subgroup versus the corresponding hazard ratios for the first level of information fraction (i.e., 25%). Figures S4 and S5 provided in the Supplementary Materials (File S) show the expected number of events and patients of the adaptive parallel subgroup-specific design and the required number of events and patients of the fixed parallel subgroup-specific design for each biomarker-defined subgroup versus the corresponding hazard ratios for the second and third level of information fraction (i.e., 50%, 75%). Figure 6 and Figures S4 and S5 show that the expected number of events for both biomarker-defined subgroups in all cases of hazard ratios is lower than the required number of events. Additionally, in all cases of interim fraction and significance levels, both the required and the expected number of events are greater in biomarker-negative subgroup as compared to the biomarker-positive subgroup. Furthermore, they show that the expected number of patients for both biomarker-defined subgroups in all cases of hazard ratios is lower than the required number of patients. In addition, in all cases of interim fraction and significance levels, both the required and the expected number of patients are greater in biomarker-negative subgroup as compared to the biomarker-positive subgroup.

## 3. Discussion

To conclude, we have considered a fixed design which evaluates the efficacy of treatment in each biomarker-defined subgroup and an adaptive approach which involves early stopping of the trial due to efficacy or futility. The scope of the simulation study of the fixed design is to investigate the power under different scenarios in a given simulation setting which takes into account accrual and follow-up of patients. Next, we extend the fixed design into a two-stage design with interim analysis used for decision making. The aim of the two-stage version was to investigate the general efficiency of the study by calculating the expected number of events and patients as well as stopping probabilities, overall power and expected duration of the study under different scenarios of the information fraction (i.e., specific proportion of the required total number of events applied in interim analysis). The tests between the two biomarker-defined subgroups are independent and one could present the results for only one of them. However, this could have added confusion regarding how control of the overall a is handled. Additionally, presenting results for both subgroups adds completeness to our study.

We programmed the simulation studies in R statistical software (codes available upon request). Our results indicate that when the information fraction used in interim analysis is low (25%) the study will not achieve adequate power. However, for equally allocated significance levels we can achieve sufficient power (between 70% and 80%) if the specific proportion of the required total number of events is equal or greater than 50%.

One significant challenge encountered when conducting such flexible trial designs is the multiplicity issues which should be carefully considered, e.g., in our simulation study we took into account the control of type I error not only because we had to combine information from both stages of the design but because we tested more than one subgroup of interest at the same time. Several methods have been proposed recently for multiplicity adjustment and suggestions of appropriate stopping boundaries when an interim analysis is introduced in the study design. Thus, the implications of the operating characteristics to the decision-making when these methods are applied should be explored to get the optimal results. Our simulation studies are limited to particular methods; however, they give us a general insight into the implications of an event driven design for which the decision making is based on the results of an interim analysis. We explore a fixed versus an adaptive approach in a popular biomarker-guided clinical trial setting which to our best knowledge has not been investigated yet.

The adaptive version of the parallel subgroup-specific design could be extended by using the sample size re-estimation approach [11]. More precisely, the idea behind this method is that at the interim analysis—which in our case will be based on a fixed percentage of target events—we can allow for re-evaluation of the sample size when there is uncertainty about the treatment effect size for which the study was powered.

Knowledge of how to design, implement and analyse biomarker-guided clinical trials is essential for testing the effectiveness of a biomarker-guided approach to treatment. The proper choice and use of such designs can increase the probability of success of clinical trials resulting in the development of novel treatments. Adaptive designs might be more complex and need more time during the planning process due to several simulations of possible scenarios that should be conducted aiming to investigate the statistical properties of the design under specific situations. However, they will continue to be an attractive approach of clinical development as they can lead to potential reduction in cost and time compared to a non-adaptive approach.

## Figures and Tables

**Table 1:** Results for expected number of events and patients, expected total study period, futility stopping probability, efficacy stopping probability and power of a two-stage design in scenario 1 of hazard ratios for different percentages of information fraction. Number of events and patients from Table 1 (calculated from Equation (1) and Equation (3) respectively) which achieve 80% power for the first scenario of hazard ratios and significance levels are also presented.

		Simulation Setting		Number		Simulated Power					
Information Fraction	Group of Patients	Significance Level	Hazard Ratio	Required Number of Events	Required Number of Patients	Expected Total Study Period (Months)	Expected Number of Events	Expected Number of Patients	Futility Stopping Probability (*FSP*)	Efficacy Stopping Probability (*ESP*)	Power
25%	Biomarker-negative	0.0125	0.6	146	168	17.6	86	99	0.3694	0.1810	0.5659
	Biomarker-positive	0.0125	0.4	45	76	16.9	25	43	0.3947	0.1894	0.5371
	Entire population	0.025	-	191	244	-	111	142	-	-	-
	Biomarker-negative	0.015	0.6	139	160	16.8	78	90	0.4172	0.1692	0.4999
	Biomarker-positive	0.010	0.4	48	81	14.0	22	38	0.5071	0.2059	0.4257
	Entire population	0.025	-	187	241	-	100	128	-	-	-
	Biomarker-negative	0.010	0.6	154	177	14.9	77	88	0.4821	0.1886	0.4454
	Biomarker-positive	0.015	0.4	43	72	16.1	23	39	0.4300	0.1885	0.4990
	Entire population	0.025	-	197	249	-	100	127	-	-	-
50%	Biomarker-negative	0.0125	0.6	146	168	21.4	106	120	0.1650	0.4114	0.7259
	Biomarker-positive	0.0125	0.4	45	76	21.4	32	54	0.1865	0.3859	0.6982
	Entire population	0.025	-	191	244	-	138	138	-	-	-
	Biomarker-negative	0.015	0.6	139	160	21.3	99	114	0.2044	0.3730	0.6697
	Biomarker-positive	0.010	0.4	48	81	19.6	31	53	0.2670	0.4279	0.6146
	Entire population	0.025	-	187	241	-	130	167	-	-	-
	Biomarker-negative	0.010	0.6	154	177	20.2	103	119	0.2435	0.4126	0.6400
	Biomarker-positive	0.015	0.4	43	72	21.0	30	50	0.2159	0.3873	0.6555
	Entire population	0.025	-	197	249	-	133	169	-	-	-
75%	Biomarker-negative	0.0125	0.6	146	168	25.0	122	140	0.0704	0.5915	0.7743
	Biomarker-positive	0.0125	0.4	45	76	24.9	37	63	0.0830	0.6036	0.7558
	Entire population	0.025	-	191	244	-	159	203	-	-	-
	Biomarker-negative	0.015	0.6	139	160	25.0	116	134	0.0943	0.5674	0.7266
	Biomarker-positive	0.010	0.4	48	81	24.2	39	65	0.1330	0.6356	0.7185
	Entire population	0.025	-	187	241	-	155	199	-	-	-
	Biomarker-negative	0.010	0.6	154	177	24.5	126	145	0.1148	0.6204	0.7273
	Biomarker-positive	0.015	0.4	43	72	24.9	36	60	0.1030	0.5708	0.7214
	Entire population	0.025	-	197	249	-	162	205	-	-	-

## Supplementary Materials

The following are available online at http://www.mdpi.com/2075-4426/7/4/19 in File S: Tables and graphical representations of simulation results, Tables S1–S4, Figures S1–S5.
