# Correction: Antoniou, M.; et al. Fixed and Adaptive Parallel Subgroup-Specific Design for Survival Outcomes: Power and Sample Size. *J. Pers. Med.* 2017, *7*, 19

**DOI:** 10.3390/jpm8020018

**Published:** 2018-05-07

**Authors:** Miranta Antoniou, Andrea L. Jorgensen, Ruwanthi Kolamunnage-Dona

**Affiliations:** 1 MRC North West Hub for Trials Methodology Research, Liverpool L69 3GL, UK; A.L.Jorgensen@liverpool.ac.uk (A.L.J.); Ruwanthi.Kolamunnage-Dona@liverpool.ac.uk (R.K.-D.); 2 Department of Biostatistics, Institute of Translational Medicine, University of Liverpool, Liverpool L69 3GL, UK

The authors wish to make the following corrections to this paper [1], due to typographical errors:

On page 6, the sentence, “A time *t_ij_* (i.e., sum of accrual time and follow-up time) is generated for each patient, and if *t_ij_* is greater than the cutoff time then it is assumed that the patient encountered the event at *t_ij_*, otherwise the patient’s event time is censored at *t_ij_*” should be “A time *t_ij_* (i.e., sum of accrual time and follow-up time) is generated for each patient, and if *t_ij_* is less than the cutoff time then it is assumed that the event was observed at *t_ij_*, otherwise the patient’s event time is censored at *t_ij_*”.

They would also like to remove the word *log rank* in page 11 in the four following cases:

“For the biomarker-negative subgroup of the two-stage adaptive design which tests the efficacy of the experimental treatment, the stopping rules are the following:

Stage 1: Reject the null hypothesis (stop for efficacy) if *T’*_1−_ ≤ ε_1−_
  Do not reject the null hypothesis (stop for futility) if *T’*_1−_ > *b*_1−_
  Continue to the second stage if ε_1−_ < *T’*_1−_ ≤ *b*_1−_

where 0 < ε_1−_ < *b*_1−_ ≤ 1 and *T’*_1−_ refers to the log-rank test statistic in the biomarker-negative subgroup at the first stage of the study,

Stage 2: Reject the null hypothesis (stop for efficacy) if *T’*_2−_ ≤ ε_2−_
  Do not reject the null hypothesis (stop for futility) if *T’*_2−_ > ε_2−_

where *T’*_2−_ refers to the log-rank test statistic in the biomarker-negative subgroup at the second stage of the study. 

For the biomarker-positive subgroup of the two-stage adaptive design which tests the efficacy of the experimental treatment, the stopping rules are the following,

Stage 1: Reject the null hypothesis (stop for efficacy) if *T’*_1+_ ≤ ε_1+_
  Do not reject the null hypothesis (stop for futility) if *T’*_1+_ > *b*_1+_
  Continue to the second stage if ε_1+_ < *T’*_1+_ ≤ *b*_1+_

where 0 < *a*_1+_ < *b*_1+_ ≤ 1 and *T’*_1+_ refers to the log-rank test statistic in the biomarker-positive subgroup at the first stage of the study,

Stage 2: Reject the null hypothesis (stop for efficacy) if *T’*_2+_ ≤ ε_2+_
  Do not reject the null hypothesis (stop for futility) if *T’*_2+_ > ε_2+_

where *T’*_2+_ refers to the log-rank test statistic in the biomarker-positive subgroup at the second stage of the study.”

These changes have no material impact on the conclusions of the paper. The authors would like to apologize for any inconvenience caused to the readers by these changes.
