# Supplementary material for: Fixed and Adaptive Parallel Subgroup-Specific Design for Survival Outcomes: Power and Sample Size
Source: J Pers Med. 2017 Dec 4;7(4):19. doi: 10.3390/jpm7040019 (PMC5748631; doi:10.3390/jpm7040019)
Supplement: Supplementary file 1 [file jpm-07-00019-s001.zip › File S.docx]

**Supplementary Material: Fixed and adaptive Parallel Subgroup-Specific design for survival outcomes: power and sample size**

**Miranta Antoniou, Andrea L Jorgensen and Ruwanthi Kolamunnage-Dona**

**File S. Tables and graphical representations of simulation results**

**Table S1.** Accrual rate and number of events and patients (calculated from (7), (1) and (3) respectively) which achieve approximate 80% power for different scenarios of hazard ratios and significance levels, and the corresponding power of each biomarker-defined subgroup yielded from the simulation.

|  | **Simulation setting** | | **Accrual rate and required numbers** | | | **Simulated power** |
| --- | --- | --- | --- | --- | --- | --- |
| **Group of patients** | **Significance level** | **Hazard ratio** | **Accrual rate** | **Number of**  **events** | **Number of**  **patients** | **Power** |
| Biomarker-negative  Biomarker-positive  Entire population | 0.0125  0.0125  0.025 | 0.6  0.4  - | 9  4  - | 146  45  191 | 168  76  244 | 0.798  0.788  - |
| Biomarker-negative  Biomarker-positive  Entire population | 0.015  0.010  0.025 | 0.6  0.4  - | 9  4  - | 139  48  187 | 160  81  241 | 0.796  0.792  - |
| Biomarker-negative  Biomarker-positive  Entire population | 0.010  0.015  0.025 | 0.6  0.4  - | 10  4  - | 154  43  197 | 177  73  250 | 0.791  0.792  - |
| Biomarker-negative  Biomarker-positive  Entire population | 0.0125  0.0125  0.025 | 0.7  0.5  - | 19  7  - | 299  79  378 | 335  126  461 | 0.804  0.786  - |
| Biomarker-negative  Biomarker-positive  Entire population | 0.015  0.010  0.025 | 0.7  0.5  - | 18  7  - | 285  84  369 | 320  133  453 | 0.798  0.793  - |
| Biomarker-negative  Biomarker-positive  Entire population | 0.010  0.015  0.025 | 0.7  0.5  - | 20  7  - | 316  76  392 | 354  120  474 | 0.798  0.800  - |
| Biomarker-negative  Biomarker-positive  Entire population | 0.0125  0.0125  0.025 | 0.8  0.6  - | 47  12  - | 764  146  910 | 841  220  1061 | 0.796  0.796  - |
| Biomarker-negative  Biomarker-positive  Entire population | 0.015  0.010  0.025 | 0.8  0.6  - | 45  13  - | 729  154  883 | 803  232  1035 | 0.800  0.796  - |
| Biomarker-negative  Biomarker-positive  Entire population | 0.010  0.015  0.025 | 0.8  0.6  - | 49  12  - | 806  139  945 | 888  210  1098 | 0.799  0.793  - |
| Biomarker-negative  Biomarker-positive  Entire population | 0.0125  0.0125  0.025 | 0.9  0.7  - | 207  24  - | 3425  299  3724 | 3720  434  4154 | 0.806  0.804  - |
| Biomarker-negative  Biomarker-positive  Entire population | 0.015  0.010  0.025 | 0.9  0.7  - | 197  25  - | 3268  316  3584 | 3550  458  4008 | 0.803  0.798  - |
| Biomarker-negative  Biomarker-positive  Entire population | 0.010  0.015  0.025 | 0.9  0.7  - | 218  23  - | 3616  285  3901 | 3928  414  4342 | 0.804  0.796  - |

**Table S2.** Results for expected number of events and patients, expected total study period, futility stopping probability, efficacy stopping probability and power of a two-stage design in scenario 2 of hazard ratios for different percentages of information fraction. Number of events and patients from Table 1 (calculated from (1) and (3) respectively) which achieve 80% power for the second scenario of hazard ratios and significance levels are also presented.

|  |  | **Simulation setting** | | **Number** | | | **Simulated power** | | | | | |
| --- | --- | --- | --- | --- | --- | --- | --- | --- | --- | --- | --- | --- |
| **Information fraction** | **Group of patients** | **Significance level** | **Hazard ratio** | **Required Number of events** | | **Required**  **Number of**  **patients** | **Expected Total study period (months)** | **Expected Number of events** | **Expected**  **Number of**  **patients** | **FSP** | **ESP** | **Power** |
| 25% | Biomarker-negative  Biomarker-positive  Entire population | 0.0125  0.0125  0.025 | 0.7  0.5  - | 299  79  378 | 335  125  460 | | 17.6  16.9  - | 176  44  220 | 197  70  267 | 0.3672  0.3931  - | 0.1817  0.1895  - | 0.5678  0.5382  - |
|  | Biomarker-negative  Biomarker-positive  Entire population | 0.015  0.010  0.025 | 0.7  0.5  - | 285  84  369 | 320  133  453 | | 16.8  14.0  - | 160  39  199 | 179  62  241 | 0.4173  0.5070  - | 0.1696  0.2060  - | 0.5000  0.4258  - |
|  | Biomarker-negative  Biomarker-positive  Entire population | 0.010  0.015  0.025 | 0.7  0.5  - | 316  76  392 | 354  121  475 | | 14.9  16.1  - | 157  41  198 | 176  65  241 | 0.4823  0.4268  - | 0.1879  0.1899  - | 0.4450  0.5028  - |
| 50% | Biomarker-negative  Biomarker-positive  Entire population | 0.0125  0.0125  0.025 | 0.7  0.5  - | 299  79  378 | 335  125  460 | | 21.7  21  - | 217  55  272 | 243  88  331 | 0.1655  0.1846  - | 0.3848  0.4142  - | 0.7246  0.7004  - |
|  | Biomarker-negative  Biomarker-positive  Entire population | 0.015  0.010  0.025 | 0.7  0.5  - | 285  84  369 | 320  133  453 | | 21.3  19.6  - | 203  55  258 | 228  87  315 | 0.2036  0.2674  - | 0.3736  0.4273  - | 0.6700  0.6145  - |
|  | Biomarker-negative  Biomarker-positive  Entire population | 0.010  0.015  0.025 | 0.7  0.5  - | 316  76  392 | 354  121  475 | | 20.2  21.0  - | 212  53  265 | 238  85  323 | 0.2425  0.2118  - | 0.4127  0.3915  - | 0.6414  0.6593  - |
| 75% | Biomarker-negative  Biomarker-positive  Entire population | 0.0125  0.0125  0.025 | 0.7  0.5  - | 299  79  378 | 335  125  460 | | 25.0  24.8  - | 249  65  314 | 280  104  384 | 0.0707  0.0824  - | 0.5922  0.6046  - | 0.7739  0.7571  - |
|  | Biomarker-negative  Biomarker-positive  Entire population | 0.015  0.010  0.025 | 0.7  0.5  - | 285  84  369 | 320  133  453 | | 25.0  24.2  - | 238  68  306 | 267  107  374 | 0.0944  0.1322  - | 0.5671  0.6366  - | 0.7264  0.7193  - |
|  | Biomarker-negative  Biomarker-positive  Entire population | 0.010  0.015  0.025 | 0.7  0.5  - | 316  76  392 | 354  121  475 | | 24.5  24.9  - | 258  63  21 | 289  100  389 | 0.1141  0.1007  - | 0.6187  0.5812  - | 0.7262  0.7277  - |

**Table S3.** Results for expected number of events and patients, expected total study period, futility stopping probability, efficacy stopping probability and power of a two-stage design in scenario 3 of hazard ratios for different percentages of information fraction. Number of events and patients from Table 1 (calculated from (1) and (3) respectively) which achieve 80% power for the third scenario of hazard ratios and significance levels are also presented.

|  |  | **Simulation setting** | | **Number** | | **Simulated power** | | | | | |
| --- | --- | --- | --- | --- | --- | --- | --- | --- | --- | --- | --- |
| **Information fraction** | **Group of patients** | **Significance level** | **Hazard ratio** | **Required Number of events** | **Required**  **Number of**  **patients** | **Expected Total study period (months)** | **Expected Number of events** | **Expected**  **Number of**  **patients** | ***FSP*** | ***ESP*** | **Power** |
| 25% | Biomarker-negative  Biomarker-positive  Entire population | 0.0125  0.0125  0.025 | 0.8  0.6  - | 764  146  912 | 841  221  1061 | 17.6  16.9  - | 449  82  531 | 495  125  620 | 0.3678  0.3921  - | 0.1815  0.1903  - | 0.5680  0.5399  - |
|  | Biomarker-negative  Biomarker-positive  Entire population | 0.015  0.010  0.025 | 0.8  0.6  - | 729  154  883 | 803  233  1036 | 16.8  14  - | 409  72  481 | 450  109  559 | 0.4162  0.5072  - | 0.1694  0.2051  - | 0.5004  0.4245  - |
|  | Biomarker-negative  Biomarker-positive  Entire population | 0.010  0.015  0.025 | 0.8  0.6  - | 806  139  945 | 888  210  1098 | 14.9  16.1  - | 400  75  475 | 440  113  553 | 0.4839  0.4276  - | 0.1881  0.1893  - | 0.4434  0.5013  - |
| 50% | Biomarker-negative  Biomarker-positive  Entire population | 0.0125  0.0125  0.025 | 0.8  0.6  - | 764  146  912 | 841  221  1061 | 21.7  21.0 | 554  102  656 | 609  155  764 | 0.1658  0.1827  - | 0.3849  0.4161  - | 0.7244  0.7034  - |
|  | Biomarker-negative  Biomarker-positive  Entire population | 0.015  0.010  0.025 | 0.8  0.6  - | 729  154  883 | 803  233  1036 | 21.3  19.6  - | 518  101  619 | 571  152  723 | 0.2036  0.2687  - | 0.3743  0.4245  - | 0.6711  0.6118  - |
|  | Biomarker-negative  Biomarker-positive  Entire population | 0.010  0.015  0.025 | 0.8  0.6  - | 806  139  945 | 888  210  1098 | 20.2  21  - | 542  97  639 | 597  147  744 | 0.2438  0.2126  - | 0.4123  0.3895  - | 0.6400  0.6576  - |
| 75% | Biomarker-negative  Biomarker-positive  Entire population | 0.0125  0.0125  0.025 | 0.8  0.6  - | 764  146  912 | 841  221  1061 | 25.1  24.8  - | 637  121  758 | 702  183  885 | 0.0706  0.0821  - | 0.5923  0.6053  - | 0.7739  0.7580  - |
|  | Biomarker-negative  Biomarker-positive  Entire population | 0.015  0.010  0.025 | 0.8  0.6  - | 729  154  883 | 803  233  1036 | 25.0  24.2  - | 608  124  732 | 670  188  858 | 0.0944  0.1345  - | 0.568  0.6336  - | 0.7276  0.7160  - |
|  | Biomarker-negative  Biomarker-positive  Entire population | 0.010  0.015  0.025 | 0.8  0.6  - | 806  139  945 | 888  210  1098 | 24.5  24.9  - | 658  115  773 | 725  175  900 | 0.1144  0.1021  - | 0.6199  0.5755  - | 0.7252  0.7236  - |

**Table S4.** Results for expected number of events and patients, expected total study period, futility stopping probability, efficacy stopping probability and power of a two-stage design in scenario 4 of hazard ratios for different percentages of information fraction. Number of events and patients from Table 1 (calculated from (1) and (3) respectively) which achieve 80% power for the fourth scenario of hazard ratios and significance levels are also presented.

|  |  | **Simulation setting** | | **Number** | | **Simulated power** | | | | | |
| --- | --- | --- | --- | --- | --- | --- | --- | --- | --- | --- | --- |
| **Information fraction** | **Group of patients** | **Significance level** | **Hazard ratio** | **Required Number of events** | **Required**  **Number of**  **patients** | **Expected Total study period (months)** | **Expected Number of events** | **Expected**  **Number of**  **patients** | ***FSP*** | ***ESP*** | **Power** |
| 25% | Biomarker-negative  Biomarker-positive  Entire population | 0.0125  0.0125  0.025 | 0.9  0.7  - | 3425  299  3724 | 3720  434  4154 | 17.6  16.9  - | 2014  168  2182 | 2187  245  2432 | 0.3678  0.3924  - | 0.1816  0.1897  - | 0.5678  0.5392  - |
|  | Biomarker-negative  Biomarker-positive  Entire population | 0.015  0.010  0.025 | 0.9  0.7  - | 3268  316  3584 | 3550  458  4008 | 16.8  14  - | 1831  147  1978 | 1989  213  2202 | 0.4171  0.5076  - | 0.1691  0.2056  - | 0.4995  0.4245  - |
|  | Biomarker-negative  Biomarker-positive  Entire population | 0.010  0.015  0.025 | 0.9  0.7  - | 3616  285  3901 | 3928  413  4341 | 14.9  16.1  - | 1794  153  1947 | 1948  222  2170 | 0.4838  0.4277  - | 0.1882  0.1892  - | 0.4436  0.5010  - |
| 50% | Biomarker-negative  Biomarker-positive  Entire population | 0.0125  0.0125  0.025 | 0.9  0.7  - | 3425  299  3724 | 3720  434  4154 | 21.7  21  - | 2482  210  2692 | 2696  304  3000 | 0.1656  0.1835  - | 0.3849  0.4151  - | 0.7248  0.7017  - |
|  | Biomarker-negative  Biomarker-positive  Entire population | 0.015  0.010  0.025 | 0.9  0.7  - | 3268  316  3584 | 3550  458  4008 | 21.3  19.6  - | 2324  206  2530 | 2524  299  2823 | 0.2046  0.2681  - | 0.3732  0.4260  - | 0.6704  0.6129  - |
|  | Biomarker-negative  Biomarker-positive  Entire population | 0.010  0.015  0.025 | 0.9  0.7  - | 3616  285  3901 | 3928  413  4341 | 20.2  21.0  - | 2430  199  2629 | 2639  289  2928 | 0.2437  0.2127  - | 0.4124  0.3894  - | 0.6401  0.6575  - |
| 75% | Biomarker-negative  Biomarker-positive  Entire population | 0.0125  0.0125  0.025 | 0.9  0.7  - | 3425  299  3724 | 3720  434  4154 | 25.0  24.8  - | 2857  248  3105 | 3103  359  3462 | 0.0709  0.0826  - | 0.5925  0.6051  - | 0.7734  0.7572  - |
|  | Biomarker-negative  Biomarker-positive  Entire population | 0.015  0.010  0.025 | 0.9  0.7  - | 3268  316  3584 | 3550  458  4008 | 25.0  24.2  - | 2727  255  2982 | 2962  370  3332 | 0.0944  0.1344  - | 0.5679  0.6338  - | 0.7272  0.7162  - |
|  | Biomarker-negative  Biomarker-positive  Entire population | 0.010  0.015  0.025 | 0.9  0.7  - | 3616  285  3901 | 3928  413  4341 | 24.5  24.9  - | 2952  237  3189 | 3206  343  3549 | 0.1146  0.1022  - | 0.6199  0.5752  - | 0.7254  0.7236  - |

| **** |
| --- |

**Figure S1.** Efficacy stopping probability, futility stopping probability and power of a two-stage design versus the interim fraction (25%, 50%, 75%) in each biomarker-defined subgroup for scenario 2 of hazard ratios. Each row of graphs represents the different probabilities versus the interim fraction of each biomarker-defined subgroup when (i) $a_{-}=a_{+}=0.0125$, (ii) $a_{-}=0.015$ and $a_{+}=0.010$ and (iii) $a_{-}=0.010$ and $a_{+}=0.015$ respectively.

| **** |
| --- |

**Figure S2.** Efficacy stopping probability, futility stopping probability and power of a two-stage design versus the interim fraction (25%, 50%, 75%) in each biomarker-defined subgroup for scenario 3 of hazard ratios. Each row of graphs represents the different probabilities versus the interim fraction of each biomarker-defined subgroup when (i) $a_{-}=a_{+}=0.0125$, (ii) $a_{-}=0.015$ and $a_{+}=0.010$ and (iii) $a_{-}=0.010$ and $a_{+}=0.015$ respectively.

| **** |
| --- |

**Figure S3.** Efficacy stopping probability, futility stopping probability and power of a two-stage design versus the interim fraction (25%, 50%, 75%) in each biomarker-defined subgroup for scenario 4 of hazard ratios. Each row of graphs represents the different probabilities versus the interim fraction of each biomarker-defined subgroup when (i) $a_{-}=a_{+}=0.0125$, (ii) $a_{-}=0.015$ and $a_{+}=0.010$ and (iii) $a_{-}=0.010$ and $a_{+}=0.015$ respectively.

| **** |
| --- |

**Figure S4.** Expected number of events and patients in two-stage design and required number of events and patients in one-stage design for each biomarker-defined subgroup versus the hazard ratios of each biomarker-defined subgroup when the interim fraction is 50%. The first two graphical representations in each row of graphs represent the number of events versus the hazard ratio of each biomarker-defined subgroup when (i) $a_{-}=a_{+}=0.0125$, (ii) $a_{-}=0.015$ and $a_{+}=0.010$ and (iii) $a_{-}=0.010$ and $a_{+}=0.015$ respectively. The remaining graphical representations in each row of graphs represent the number of patients versus the hazard ratio of each biomarker-defined subgroup when (i) $a_{-}=a_{+}=0.0125$, (ii) $a_{-}=0.015$ and $a_{+}=0.010$ and (iii) $a_{-}=0.010$ and $a_{+}=0.015$.

| **** |
| --- |

**Figure S5.** Expected number of events and patients in two-stage design and required number of events and patients in one-stage design for each biomarker-defined subgroup versus the hazard ratios of each biomarker-defined subgroup when the interim fraction is 75%. The first two graphical representations in each row of graphs represent the number of events versus the hazard ratio of each biomarker-defined subgroup when (i) $a_{-}=a_{+}=0.0125$, (ii) $a_{-}=0.015$ and $a_{+}=0.010$ and (iii) $a_{-}=0.010$ and $a_{+}=0.015$ respectively. The remaining graphical representations in each row of graphs represent the number of patients versus the hazard ratio of each biomarker-defined subgroup when (i) $a_{-}=a_{+}=0.0125$, (ii) $a_{-}=0.015$ and $a_{+}=0.010$ and (iii) $a_{-}=0.010$ and $a_{+}=0.015$.
